# Lectin Complement Protein Collectin 11 (CL-K1) and Susceptibility to Urinary Schistosomiasis

**DOI:** 10.1371/journal.pntd.0003647

**Published:** 2015-03-25

**Authors:** Justin S. Antony, Olusola Ojurongbe, Peter G. Kremsner, Thirumalaisamy P. Velavan

**Affiliations:** 1 Institute of Tropical Medicine, University of Tübingen, Tübingen, Germany; 2 Ladoke Akintola University of Technology, Ogbomoso, Nigeria; 3 Fondation Congolaise pour la Recherche Medicale, Brazzaville, Republic of Congo; Centers for Disease Control and Prevention, UNITED STATES

## Abstract

**Background:**

Urinary Schistosomiasis is a neglected tropical disease endemic in many sub Saharan -African countries. Collectin Kidney 1 (CL-K1, encoded by *COLEC11* on chromosome 2p25.3), a member of the vertebrate C-type lectin super family, has recently been identified as pattern-recognition molecule (PRR) of the lectin complement pathway. CL-K1 is preferentially expressed in the kidneys, but also in other organs and it is considered to play a role in host defense to some infectious agents. Schistosome teguments are fucosylated and CL-K1 has, through its collagen-like domain, a high binding affinity to fucose.

**Methodology/Principal Findings:**

We utilized a Nigerian study group consisting of 167 *Schistosoma haematobium* infected individuals and 186 matched healthy subjects, and investigated the contribution of CL-K1 deficiency and of *COLEC11* polymorphisms to infection phenotype. Higher CL-K1 serum levels were associated with decreased risk of schistosome infection (*P^corr^* = 0.0004). CL-K1 serum levels were differentially distributed between the *COLEC11* genotypes and haplotypes observed. The non-synonymous variant *p*.*R216H* was associated with the occurrence of schistosomiasis (OR = 0.44, 95%CI = 0.22–0.72, *P^corr^* = 0.0004). The reconstructed COLEC11*TCCA haplotypes were associated with higher CL-K1 serum levels (*P* = 0.002) and with decreased schistosomiasis (OR = 0.38, 95%CI = 0.23–0.63, *P^corr^* = 0.0001).

**Conclusions:**

In agreement with findings from our earlier published study, our findings support the observation that CL-K1 and their functional variants may be host factors associated with protection in schistosomiasis and may be a useful marker for further investigations.

## Author Summary

Collectin Kidney 1 (CL-K1) was discovered in 2006. It is a pattern-recognition molecule (PRR) of the lectin complement pathway and is mainly expressed in the kidneys. We investigated a possible functional role of CL-K1 during urinary schistosomiasis on the basis of the rationale: i). Schistosome teguments are fucosylated and CL-K1 has high binding affinity to fucose ii). As CL-K1 is structurally similar to MBL, we hypothesize the involvement of CL-K1 in immune modulation during urinary schistosomiasis. We investigated the circulating CL-K1 serum levels in our study group. We initially screened the entire promoter and the exons of the *COLEC11* gene for population specific variants. We validated four promoter variants and one non-synonymous substitution in exon8 for possible associations with urinary schistosomiasis and circulating CL-K1 serum levels. The study was conducted in Nigeria where the largest number of registered cases of schistosomiasis (29 million) for any sub-Saharan African country has been documented. Our findings support the observation that CL-K1 and functional variants are host factors that may be associated with protection in schistosomiasis and may be a useful marker for further investigations.

## Introduction

Urogenital schistosomiasis, which is caused by infection with the trematode *Schistosoma haematobium*, is a major public health problem in sub-Saharan Africa (SSA). Of the more than 200 million cases reported worldwide, 93% occur in SSA [1]. Up to two-thirds of *S*. *haematobium* infections result in genital schistosomiasis [2]. The incidence of *S*. *haematobium* infections in SSA, however, is most likely underreported and might be much higher [3]. Schistosomiasis accounts for the loss of more than 70 million disability adjusted life years (DALYs) [4,5]. A large proportion of infected individuals experience hematuria (70 million), dysuria (32 million), bladder-wall pathology (18 million), and severe hydronephrosis (10 million) [6]. Urinary schistosomiasis is endemic in Nigeria and approximately 25 million people are currently infected, with an estimated 101 million at risk [7]. Schistosomiasis can also increase the risk of urinary tract infections and bladder cancer [8–10]. Children and early adolescents are at high risk of infection as their daily activities regularly include contact with water infested with infectious cercariae [11]. Limited access to praziquantel treatment for schistosomiasis, repeated re-exposure, and rapid reinfections all contribute to the disease burden [11,12].

Schistosomes are bisexual multicellular helminth parasites with six developmental stages including, adult worms, eggs, miracidia, sporocysts, cercariae and schistosomulae [13]. Schistosomes have an outer syncytial cytoplasmic layer, the tegument [14]. Previous studies have shown that the teguments consist of fucosylated carbohydrate epitopes (glycotopes) [15] and glycoproteins [16] which are expressed at all developmental stages. These glycoconjugates act as pathogen associated molecular patterns (PAMPs) that are recognized by pattern recognition molecules (PRMs) such as the C-type lectins [17]. Earlier *in vitro* studies have demonstrated successful complement-mediated tegument damage in the adult schistosomes [18]. We have previously shown that the lectin proteins mannose binding lectin (MBL) and MBL-associated serine protease 1 (MASP-1) and MASP-2 interact with schistosomal glycoconjugates, and subsequently activate the lectin complement cascade [19].

Collectin kidney 1 (CL-K1 also known as Collectin 11), is a member of the group of C-type lectins. The role of CL-K1 appears to be analogous to that of other C-type lectins [20]. To date, many studies have concentrated on the complement proteins MBL [21–24] and ficolins [25–28] in disease. Far less is known about the mechanism of action of CL-K1. The human CL-K1 is encoded by *COLEC11* (OMIM 612502) on chromosome 2 at position 2p25.3 [29]. CL-K1 is a circulating serum protein and is expressed in many tissues. High mRNA expression is observed in kidneys, liver and in the adrenal glands. Similar to MBL, CL-K1 has a collagen like domain and a carbohydrate recognition domain (CRD) [20,30,31]. Six different genetic variants have been observed in a homozygous state in individuals affected with the rare Carnevale, Mingarelli, Malpuech and Michels (also known as 3MC) syndrome [32]. Two affected individuals with the p.Gly204Ser amino acid substitution in CRD of *COLEC11* had undetectable amounts of CL-K1 in their serum. Moreover, CL-K1 was shown to be a guidance cue for neural cell migration during embryogenesis [32]. The *COLEC11* variant rs10210631 is responsible for high IgE production in children [33]. CL-K1 recognizes pathogens by interacting with parasites' glycoconjugates [20,30]. A recent study has shown that CL-K1 can also deposit C4b upon binding with mannan in the presence of MASP-2 [34]. In addition, interactions of CL-K1 with MASP-1/3 have been well demonstrated [20,30].

We investigated the functional role of CL-K1 during urinary schistosomiasis on the basis of the following rationale: i). Schistosome teguments are fucosylated and CL-K1 has a high binding affinity to fucose [35] ii). as CL-K1 is structurally similar to MBL, we hypothesized that CL-K1 might be involved in immune modulation during urinary schistosomiasis. We therefore investigated CL-K1 serum levels in a study group of Nigerian individuals of Yoruba ethnicity as described in our previous studies [36,37]. Furthermore, we screened the entire *COLEC11* gene for population specific functional variants. We then evaluated four promoter variants and one non-synonymous substitution in *COLEC11* exon8 for associations with urinary schistosomiasis and circulating CL-K1 serum levels.

## Materials and Methods

### Ethics statement

Informed oral consent in the local language was obtained from all participants; for those who were children, informed consent was obtained from respective parents and/or guardians. The consent was verbal because the study was conducted in rural communities where the level of literacy was low. If the participants could not read or write, verbal consent was obtained after explaining the purpose of the study to them. The consent was written in a note book and only consenting individuals were recorded. The procedure was approved by the ethical committee of the Ladoke Akintola University of Technology, Ogbomoso, Nigeria. Only those who provided their consent were recruited in the study. Ethical approval was also obtained from the ethical committee of Ministry of Health, Abeokuta Ogun State, Nigeria.

### Study design

Two villages known to be endemic for *S*. *haematobium*, Ilewo Orile (Abeokuta North) and Ore (Osogbo), were chosen for the study. The communities lack sufficient clean water supply, safe waste disposal and essential health centers. Members of the communities depend on rivers in close proximity for their daily needs (collecting water, washing clothes, bathing). Fishing and petty trading are the most common occupations. Based on responses to a questionnaire, over 93% of the participants had regular water contact (defined as at least four contacts to the infected river in a week) for their daily needs. Most of the study participants had similar frequencies of exposure with water infested with infectious cercariae. Epidemiological studies of urinary schistosomiasis in Ogun state, Nigeria reported an infection rate of more than 80% [38]. The current study is a cross-sectional study and individuals were recruited blindly irrespective of their infection status. Individuals from all age groups who gave their consent to participate in the study procedure were enrolled. Urine samples were collected from all individuals and were microscopically examined. Based on the results of the microscopic examination, the participants were divided into the case group positive for *S*. *haematobium* eggs in urine (SEP) and the negative control group. The control group was further screened to determine total anti-schistosoma IgG antibodies. Based on total IgG results, the control group was divided into two subgroups. The first subgroup contained individuals positive for anti-*Schistosoma* total IgG antibodies and negative for eggs in urine (SELP), and the second subgroup was negative for anti-*Schistosoma* total IgG antibodies and negative for eggs in urine (SELN). This classification is essential in endemic areas in order to differentiate individuals who were potentially resistant from those with a previous or a current infection. Therefore, the detection of anti-*Schistosoma* total IgG was employed as a marker of exposure.

### Sample collection

Ten ml of urine were collected in a sterile container from all participants and the sample was centrifuged at 5000 rpm for 5 minutes. The supernatant was discarded and the sediment was transferred to a clean glass slide which was microscopically examined for the presence of *S*. *haematobium* eggs. For negative individuals, urine samples were collected on three successive days in order to confirm that they were true negatives. A Combur-Test reagent strip (Roche Diagnostics GmbH Mannheim, Germany) was used according to the manufacturer's procedure to estimate the degree of haematuria and proteinuria. About 5 ml of blood sample was collected from all study participants for serological assays and subsequent DNA extraction. Those positive for urinary schistosomiasis were treated with a single dose of 40mg/kg praziquantel. Stool samples were collected from all participants and processed using the Kato-Katz method in order to exclude any individuals with *S*. *mansoni* infection.

### Serological assays

Classification of the control group is essential in schistosoma endemic areas, as it is difficult to differentiate individuals without a current infection (either individuals are less susceptible or not been exposed to infection). Therefore detection of anti-schistosoma total IgG was employed as diagnostic exposure marker. Serological assays were carried out to determine the level of anti-*Schistosoma* total IgG antibodies in study participants’ sera by an in-house ELISA assay. In brief: For each individual sample, eight wells were used. The *Schistosoma mansoni* adult antigen was serially diluted from 0.02 to 2.5 μg per 200μl in carbonate buffer (NaHCO_3_ + Na_2_CO_3_ in water, pH = 9.6) and were pre-coated in each well. Negative and positive control plasma samples, were diluted 1:100 with milk buffer. 200μl of the test samples and controls were dispensed into the wells and were incubated for one hour at room temperature. After incubation and subsequent washing steps, each well was treated with 200μl of conjugate solution [goat anti-human IgG bound to alkaline phosphatase, (Sigma-Aldrich, Munich, Germany)] at a concentration of 1:10,000 in 1% milk buffer and incubated for one hour at room temperature. After incubation and subsequent washing steps (5x), 200μl of the substrate [pNPP: 4- Nitrophenyl phosphate disodium salt hexahydrate (Sigma-Aldrich, Munich, Germany)] was added to each well and further incubated for 15 minutes at room temperature. The optical density (OD) was measured at 405 nm. CL-K1 serum levels were determined in all study subjects in 1:5 diluted plasma by a commercially available CL-K1 ELISA kit ELISA kit (EIAab Science, Taiwan) following the manufacturer’s instruction. The lower detection limit of the assay was 7.8 ng/ml.

### *COLEC11* genotyping

Genomic DNA was extracted from blood cells using the QIAamp DNA mini blood kit (Qiagen, Hilden, Germany) according to the manufacturer’s instructions. In a first step, the *COLEC11* gene was screened by amplifying the promoter and the eight exons including intron-exon boundaries in 65 healthy individuals. A total of nine genomic fragments of the entire *COLEC11* promoter region, including the eight exons were amplified using 10 ng of genomic DNA. The PCR mix consisted of 1x PCR buffer (20 mM Tris-HCl, pH 8.4; 50 mM KCl, 2 mM MgCl_2_), 0.125 mM of dNTPs, 2 μM of sequence-specific primer pairs and 1 U Taq DNA polymerase (Qiagen, Hilden, Germany). A PTC-200 Thermal cycler (MJ Research, USA) was used. Primer sequences and thermal cycling parameters for the nine PCR reactions are listed in the S1 Table. Subsequently, PCR products were purified (Exo-SAP-IT; USB, Affymetrix, USA) and 1 μl of the product was used as template for DNA-sequencing (BigDye terminator v.1.1 cycle sequencing kit, Applied Biosystems, USA) on an ABI 3130XL sequencer. Sequences were aligned with the reference sequence of the *COLEC11* gene (NCBI; NG_031954.1) using the CodonCode Aligner 4.0 software (http://www.codoncode.com/) and confirmed visually from their electropherograms.

Based on observed frequencies of ≥ 10% of *COLEC11* variants and on variants with recognized functional significance, the variants subjected to further investigation were selected. Four promoter variants (−676T>C, −472T>C, −469C>G, −276C>T), and the non-synonymous (ns) substitution p.R216H in exon8 were genotyped in the entire study group using the primer pairs and PCR conditions as described in S2 Table.

### Statistical analysis

Data were analyzed using the STATA software (STATA Corp., College Station, TX, USA) and the level of significance was set to a p-value of <0.05. Kruskal Wallis rank sum tests following Dunn’s multiple comparison post test were used to analyze the correlation of serum CL-K1 levels with distinct *COLEC11* variants using Graphpad Prism v6.0. Fisher’s exact test and logistic regression analyses after adjustment for age and gender were performed to examine associations of CL-K1 variants with schistosomiasis. Correlation analyses were performed by non-parametric Spearman´s rank coefficient tests as implemented in Graphpad Prism v.6.0. Genotype and haplotype frequencies were analyzed by gene counting and expectation-maximum (EM) algorithms and the significance of deviation from Hardy-Weinberg equilibrium was tested using the random-permutation procedure as implemented in the Arlequin v. 3.5.1.2 software (http://lgb.unige.ch/arlequin). Linkage disequilibrium (LD) analysis was performed using Haploview v. 3.2 (http://broadinstitute.org/haploview).

## Results

### Characteristics of the study groups

After parasitological and serological tests, our study group was divided into three groups. The case group (SEP) was defined as being positive for *Schistosoma haematobium* eggs in urine [(n = 167), 100(60%) children, 93(55%) males, 74(45%) females and the mean age 17.5 ±13.2]. The first control group is defined as negative for *S*. *haematobium* eggs in urine but positive for anti-schistosoma total IgG antibodies (SELP) [(n = 119) 22(19%) children, 60(50%) males, 59(50%) females and the mean age 34.3±19.1] and the second control group defined as negative for *S*. *haematobium* egg in urine and also negative for anti-schistosoma total IgG antibodies (SELN) [(n = 67), 33(49%) children, 41(61%) males, 26(39%) females and the mean age 20.4 ±17.1]. All of the study subjects belong to the Yoruba ethnicity of the Nigerian population. The epidemiological data for the case control groups were: Mean age (SEP = 17.5 [4–71], SELP = 34[4–75], SELN = 20[4–71]) and hematuria (SEP = 90%, SELP = 2.4%, SELN = 0%), respectively. The mean parasite count for individuals in the SEP group was 1595 (20–27000) per 10 ml urine.

### *COLEC11* gene polymorphisms

A two step approach was followed to obtain population specific frequencies of *COLEC11* variants in the investigated study group. First, the entire promoter region and the eight exons including the intron-exon boundaries were screened in healthy controls (SELN n = 67). The observed frequencies of variants was compared with available HapMap data (S2 Table). The LD plot of observed variants in the entire *COLEC11* gene is given in S1 Fig. In the second step, all polymorphisms in the promoter region and the non-synonymous variant p.R216H in exon8 were chosen for further genotyping in the entire study group for investigation of genetic associations with infection phenotype.

### *COLEC11* gene polymorphisms and *S*. *haematobium* infection risk

**Fig 1:**
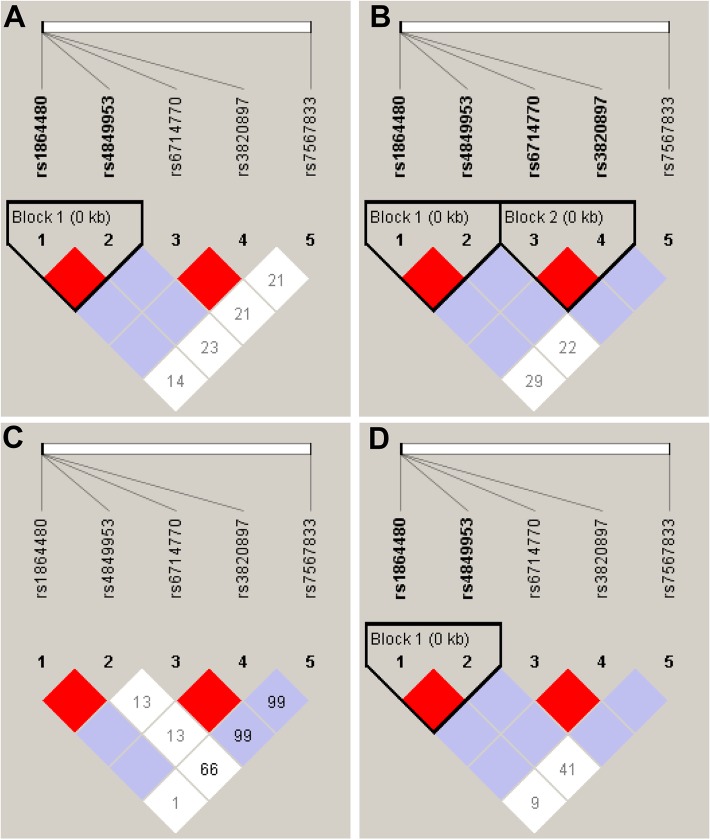
Linkage disequilibrium (LD) pattern of *COLEC11* variants in SEP cases group (A), in SELP control group (B), in SELN control group (C) and in SELP+SELN combined control group (D). Open white squares indicate a high degree of LD (D’ = 1) between pairs of markers. Numbers indicate the D’ value expressed as a percentile. The red square indicates pairs in strong LD with LOD scores ≥ 2; purple squares, D’ = 1 with LOD scores ≤ 1. The haplotype block is outlined by a solid line.

The four gene variants in the promoter region, −676T>C, −472T>C, −469C>G, −276C>T, and the non-synonymous substitution p.R216H in exon8 were genotyped. LDs of the five *COLEC11* variants in the case group and the two control subgroups are illustrated in Fig. 1. The promoter variants rs1864480 (−676T/C) and rs4849953 (−472T/C) were observed to be in strong LD in all groups. Genotype and allele frequencies in all groups were in Hardy-Weinberg equilibrium, except for variant rs1864480 (−676T/C) in the egg positive (SEP) cases.

The non-synonymous *COLEC11* variant rs7567833G/A (p.R216H) in exon8 was observed more frequently among infected individuals (SEP) than egg and IgG-negative (SELN) healthy controls. The *COLEC11* homozygous genotype of the major allele rs7567833-GG was observed significantly more often in the SEP group compared to SELN controls after adjusting for age and gender (OR = 2.35, 95%CI = 1.26–4.37,*P* ^*corr*^ = 0.004), suggesting an association with increased risk of infection. The *COLEC11* homozygous genotype of the minor allele rs7567833-AA was observed significantly less often in the SEP group than in the SELN group (OR = 0.2, 95%CI = 0.08–0.90,*P* ^*corr*^ = 0.01), showing an association with decreased risk of urinary schistosomiasis. Similar effects were also observed in the allele distributions (allelic model: OR = 0.44, 95%CI = 0.22–0.72, *P* ^*corr*^ = 0.0004; dominant model: OR = 0.42, 95%CI = 0.22–0.79, *P* ^*corr*^ = 0.0048; and recessive model: OR = 0.2, 95%CI = 0.08–0.9, *P* ^*corr*^ = 0.01) (Table 1). The observations from the different models indicate a significant contribution of the non-synonymous p.216H substitution as a host genetic factor predisposing to schistosomiasis. Variant p.216H was not observed in linkage with any other *COLEC11* variant (Fig. 1). The other investigated *COLEC11* variants −472T>C, −469C>G, −276C>T were not associated with urinary schistosomiasis.

**Table 1:** Distribution of *COLEC11—*rs7567833G/A (*p*.*R216H*) genotypes and allele(s).

** **	**Genotype**	**SEP** ^a^ **n = 167 (%)**	**SELP** ^b^ **n = 119 (%)**	**SELN** ^c^ **n = 67(%)**	**SELP** ^b^ **+SELN** ^c^ **n = 186 (%)**	**SEP** ^a^ **vs. SELN** ^c^ **OR (95% CI)**	***p*** ^*#*^ **value**
**Exon8 rs7567833 G/A (p.R216H)**	GG	114 (68.2)	81 (68)	32 (47.8)	113 (60.7)	2.35 (1.26–4.37)	0.004
	GA	46 (27.5)	37 (31)	26 (38.8)	63 (33.9)		
	AA	7 (4.3)	1 (1)	9 (13.4)	10 (5.4)	0.2 (0.08–0.9)	0.01
	**Allele**						
	G	274 (82)	199 (83.6)	90 (67.2)	289 (77.7)	**Reference**	
	A	60 (18)	39 (16.4)	44 (32.8)	83 (22.3)	0.44 (0.27–0.72)	0.0008
	**Dominant**						
	GG	114 (68.2)	81 (68)	32 (47.8)	113 (60.7)	**Reference**	
	GA+AA	53(31.8)	38(32)	35(52.2)	73 (39.3)	0.42 (0.22–0.79)	0.004
	**Recessive**						
	GG+GA	160 (95.7)	118 (99)	58 (86.4)	176 (94.6)	**Reference**	
	AA	7 (4.3)	1 (1)	9 (13.4)	10 (5.4)	0.2 (0.08–0.9)	0.01

**Note**. CI, confidence interval; OR, odds ratio.

Percentage may not add up to 100 due to rounding errors

^#^ Adjusted *P* values for age and gender

^a^ diagnosed with *S*. *haematobium* egg in urine [SEP]

^b^ Negative for *S*. *haematobium* egg in urine but positive for anti-schistosoma total IgG [SELP]

^c^ Negative for *S*. *haematobium* egg and anti-schistosoma total IgG [SELN]

The distribution of the reconstructed *COLEC11* haplotypes including −472T>C, −469C>G, −276C>T and +48912G>A are summarized in Table 2. Six haplotypes associated with circulating levels of CL-K1 were observed. Among them, COLEC11*TCCG, COLEC11*CCCG and COLEC11*TCCA were observed at higher frequencies in the entire study group. The COLEC11*TCCG haplotype, representing all major alleles, was observed more frequently in cases than in SELN controls (OR = 1.76, 95%CI = 1.15–2.70, *P* ^*corr*^ = 0.007). COLEC11*TCCA-p.R216H, was observed more frequently among SELN controls (SEP vs. SELN: OR = 0.38, 95%CI = 0.23–0.63, *P* ^*co*rr^ = 0.0001; SEP vs. SELP+SELN controls: OR = 0.66, 95%CI = 0.43–0.99, *P* ^*corr*^ = 0.04).

**Table 2:** Distribution of *COLEC11* haplotypes in the investigated study groups

COLEC11* Haplotypes	**SEP** ^a^ **(n = 334)**	**SELP** ^b^ **(n = 238)**	**SELN** ^c^ **(n = 134)**	**SELP** ^b^ **+ SELN** ^c^ **(n = 372)**	**SEP** ^a^ **vs SELN** ^c^ **OR (95% CI)**	***p*** ^#^ **value**
COLEC11*TCCG	184 (55)	142 (60)	55 (41)	197 (53)	1.76(1.15–2.70)	0.007
COLEC11*CCCG	83 (24.9)	45 (19)	33 (24.6)	78 (21)		NS
COLEC11*TCCA	50 (15)	36 (15)	42 (31.4)	78 (21)	0.38(0.23–0.63)	0.0001
COLEC11*TGTG	7 (2)	12(5)	2 (1.5)	14 (3.5)		NS
COLEC11*TGTA	6 (1.8)	0 (0)	0 (0)	0 (0)		NS
COLEC11*CCCA	4 (1.3)	3(1)	2 (1.5)	5 (1.5)		NS
**Low Expression**						
COLEC11*TCCG	184 (55)	142 (60)	55 (41)	197 (53)	1.76(1.15–2.70)	0.007
**High Expression**						
COLEC11*CCCG + COLEC11*TCCA	133 (39.8)	81 (34)	75 (56)	156 (42)	0.52(0.33–0.79)	0.002

Note. CI, confidence interval; OR, odds ratio.

Percentage may not add up to 100 due to rounding errors

^#^ Adjusted *P* values for age and gender

^a^ diagnosed with *S*. *haematobium* egg in urine [SEP]

^b^ Negative for *S*. *haematobium* egg in urine but positive for anti-schistosoma total IgG [SELP]

^c^ Negative for *S*. *haematobium* egg and anti-schistosoma total IgG [SELN]

### CL-K1 serum levels and *S*. *haematobium* infection risk

**Fig 2:**
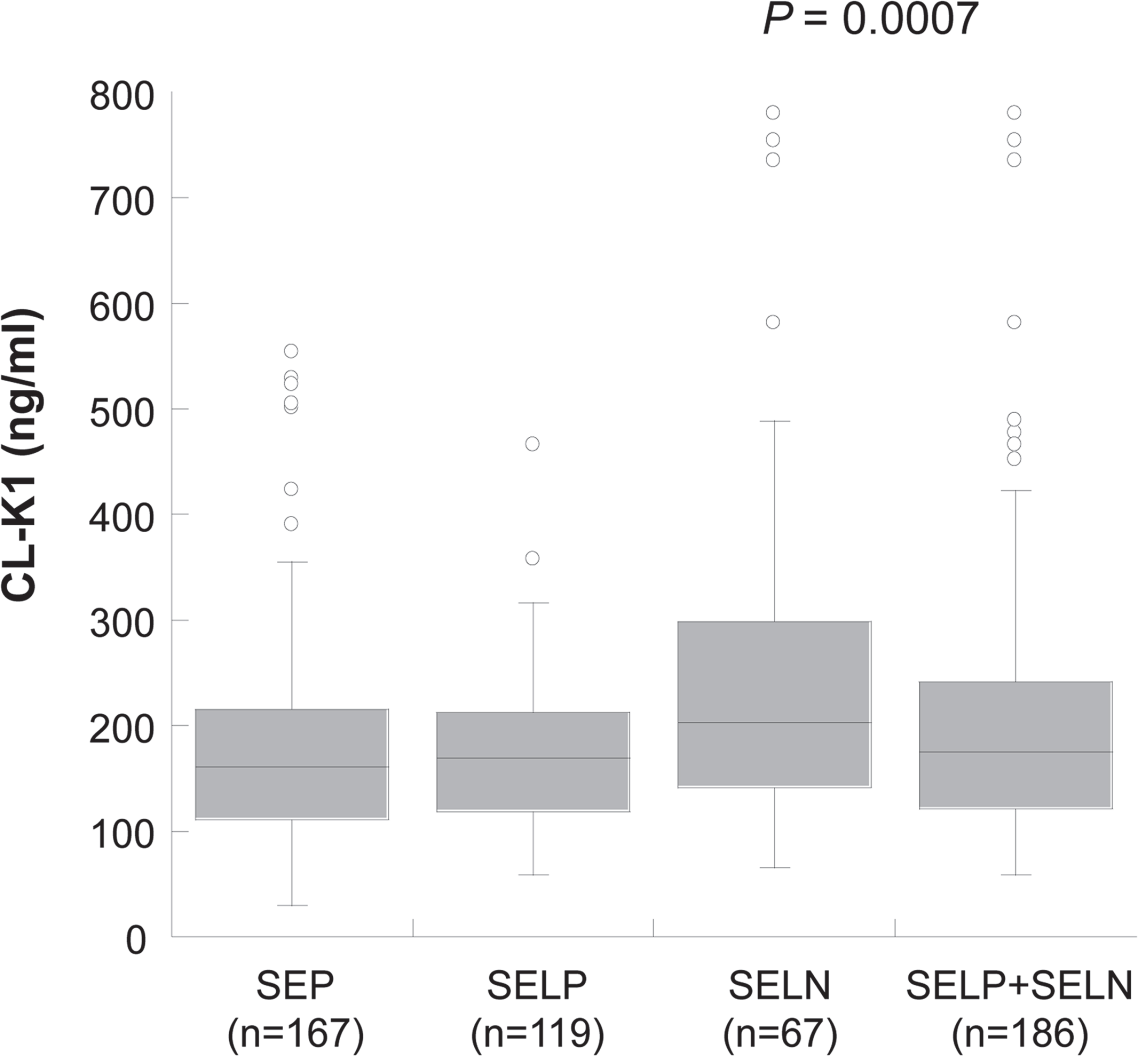
Distribution of CL-K1 serum levels (median values) among the study groups (SEP: diagnosed with *S*. *haematobium* egg in urine; SELP: Negative for *S*. *haematobium* egg in urine but positive for anti-schistosoma total IgG; SELN: Negative for *S*. *haematobium* egg and anti-schistosoma total IgG). *P* = 0.007 illustrated in the figure is calculated by Kruskal-Wallis rank sum test. Study group comparison were calculated by Dunn’s multiple comparison post test (SEP vs. SELN, *P<*0.001; SEP vs. SELP+SELN, *P*>0.05). Numbers in parentheses indicates absolute counts of sample size in each group.

Mean circulating CL-K1 serum levels among Nigerian individuals without schistosomiasis were 246±155 ng/mL, largely similar to levels observed in Japanese (340±130 ng/mL), Danish (284±∼180 ng/mL) and American populations (265±177 ng/mL) [25, 26, 35]. The median circulating CL-K1 serum levels in SEP, SELN and SELP+SELN were 161 ng/ml, 206 ng/ml and 175 ng/ml, respectively. Circulating CL-K1 serum levels were heterogeneously distributed between our study subgroups (*P* = 0.0007) (SEP vs. SELN, *P*<0.001; SEP vs. SELP+SELN, *P* >0.05) (Fig. 2).

### Association of *COLEC11 variants* to circulating CL-K1 serum levels

**Fig 3:**
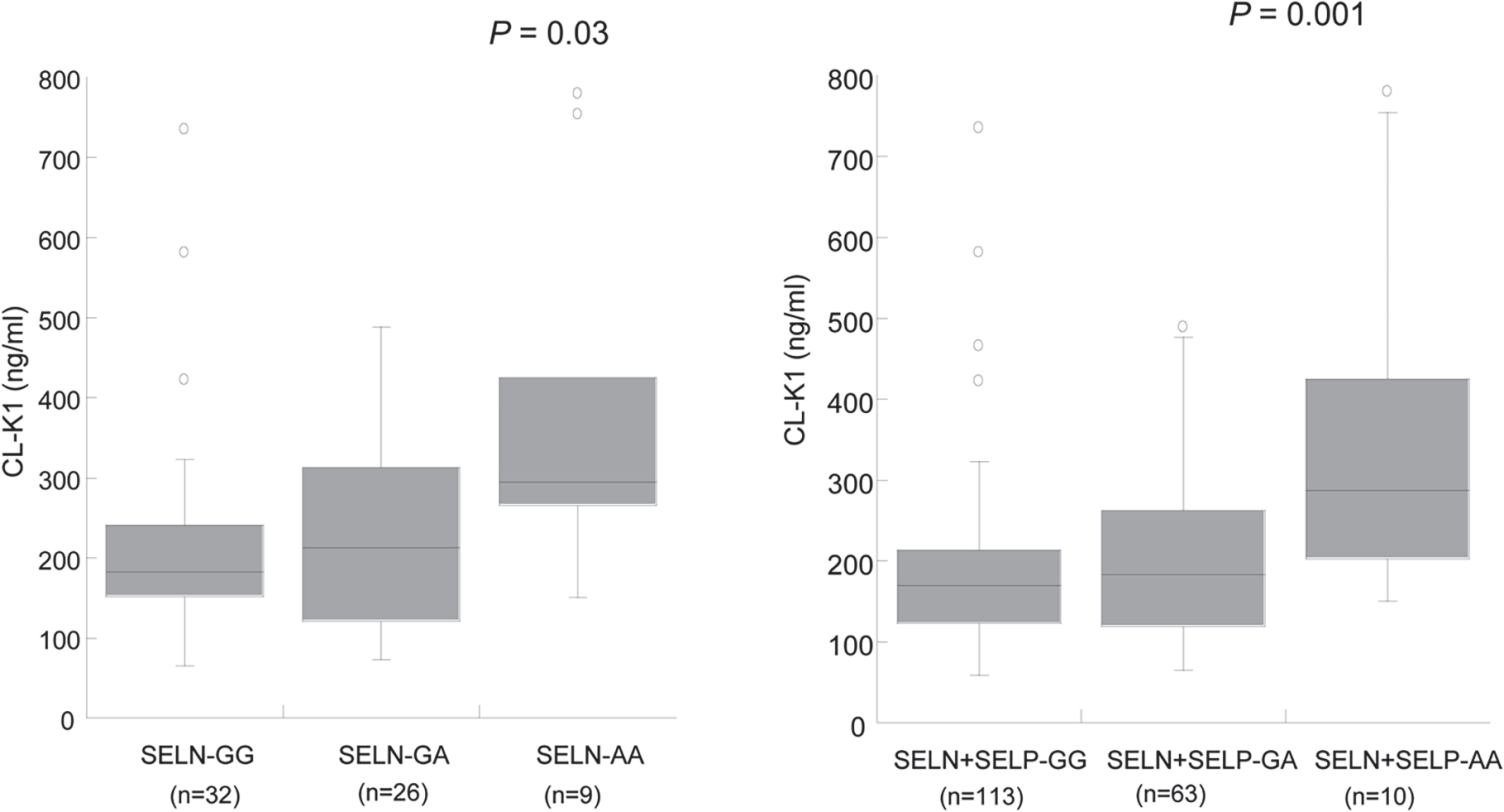
Distribution of CL-K1 serum levels (median values) with investigated rs7567833G/A (*p*.*R216H*) variant. (Left) SELN group (Right): SELP+SELN group. *P* = 0.03 and *P* = 0.001 illustrated in the figures are calculated by Kruskal-Wallis rank sum test. Study group comparison were calculated by Dunn’s multiple comparison post test (SELN-GG vs. SELN-AA, *P<*0.05; SELN-GA vs. SELN-AA, *P*>0.05); (SELN+SELP-GG vs. SELN+SELP-AA, *P*<0.01; SELN+SELP-GA vs. SELN+SELP-AA, *P*<0.05). Numbers in parentheses indicates absolute counts of sample size in each group. (**SEP**: diagnosed with *S*. *haematobium* egg in urine; **SELP**: Negative for *S*. *haematobium* egg in urine but positive for anti-schistosoma total IgG; **SELN**: Negative for *S*. *haematobium* egg and anti-schistosoma total IgG).

**Fig 4:**
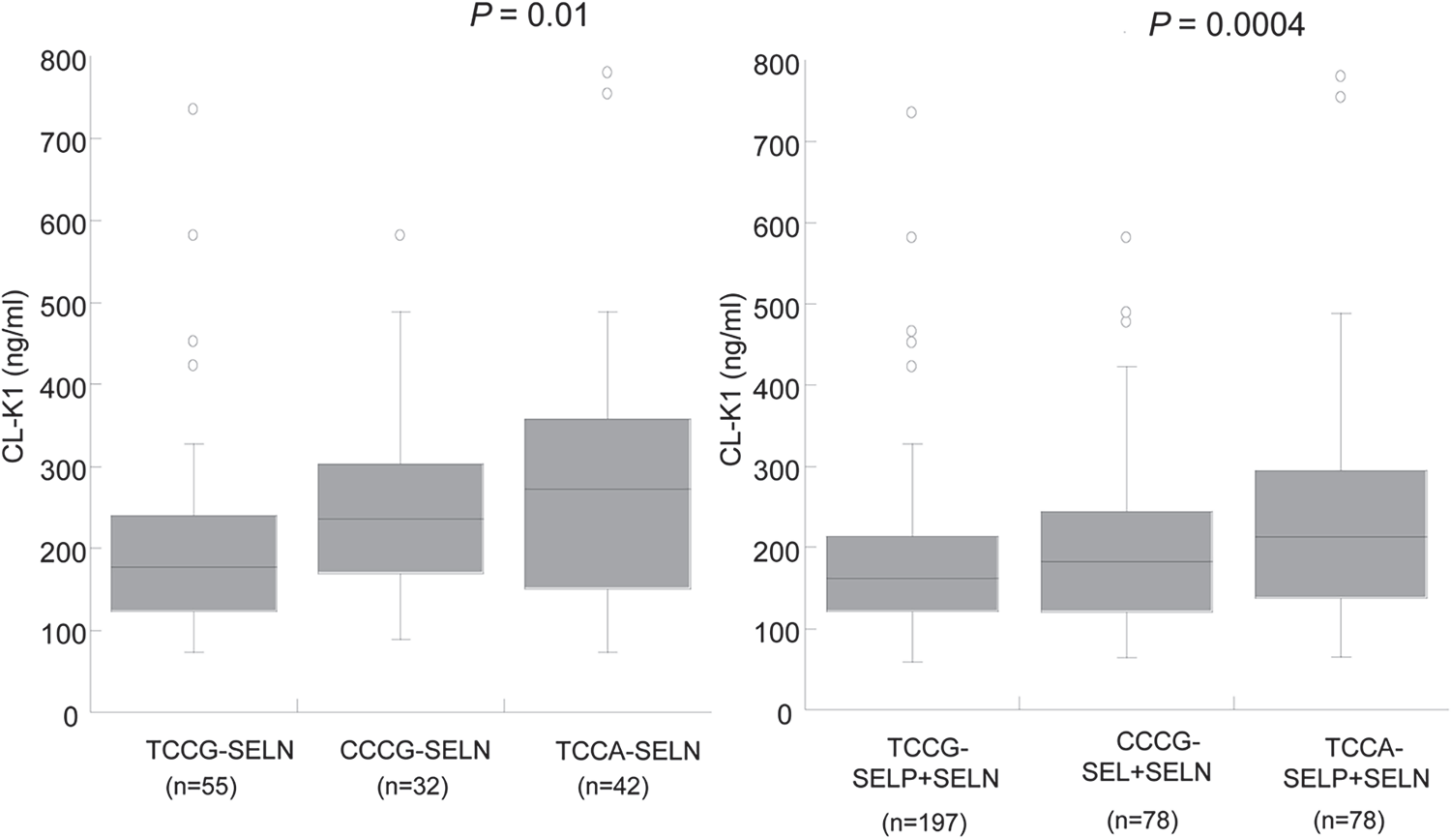
Distribution of CL-K1 serum levels (median values) with investigated *COLEC11* haplotypes (Left) SELN controls (Right): SELP+SELN combined controls. *P* = 0.03 and *P* = 0.0004 illustrated in the figures are calculated by Kruskal-Wallis rank sum test. Study group comparison were calculated by Dunn’s multiple comparison post test (COLEC11*TCCG-SELN vs. COLEC11*TCCA-SELN, *P<*0.05; COLEC11*CCCG-SELN vs. COLEC11*TCCA-SELN, *P>0*.*05*; COLEC11*TCCG-SELN+SELP vs. COLEC11*TCCA-SELN+SELP, *P<*0.001; COLEC11*TCCG-SELN+SELP vs. COLEC11*CCCG-SELN+SELP, *P>*0.05). Numbers in parentheses indicate absolute counts of sample size in each group. **SEP**: diagnosed with *S*. *haematobium* egg in urine; **SELP**: Negative for *S*. *haematobium* egg in urine but positive for anti-schistosoma total IgG; **SELN**: Negative for *S*. *haematobium* egg and anti-schistosoma total IgG).

**Fig 5:**
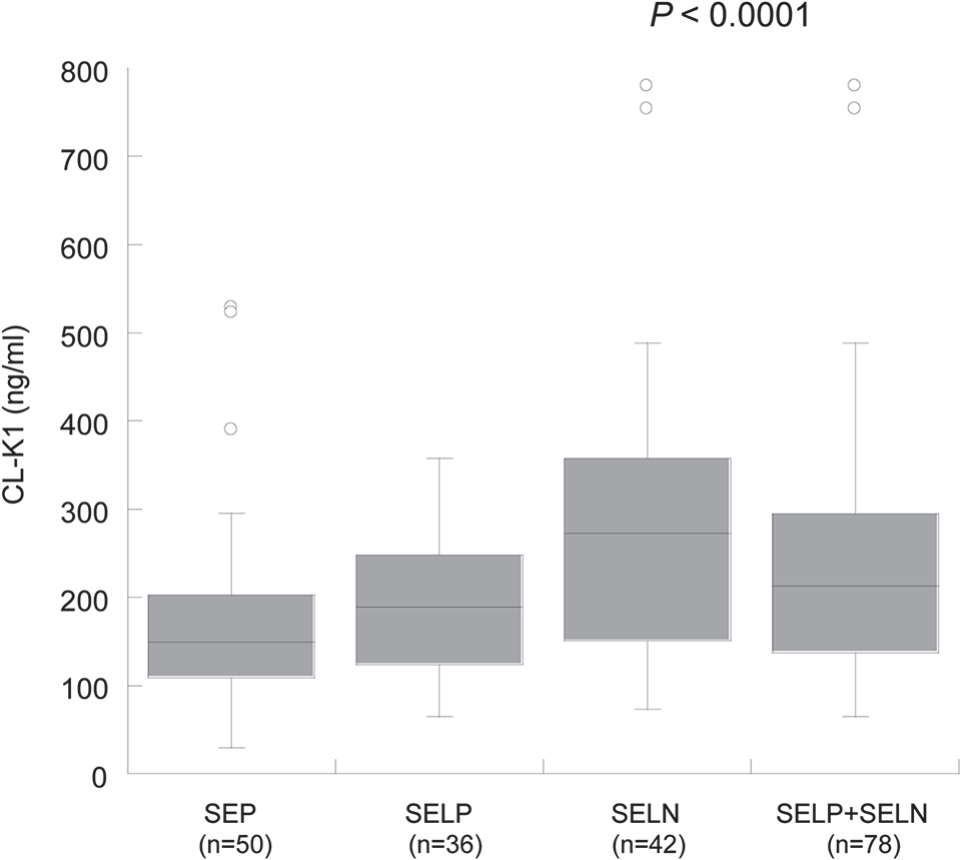
Distribution of CL-K1 serum levels (median values) with investigated COLEC11*TCCA haplotype in all study groups. *P*<0.0001 illustrated in the figure is calculated by Kruskal-Wallis rank sum test. Study group comparison were calculated by Dunn’s multiple comparison post test (COLEC11*TCCA-SEP vs. COLEC11*TCCA-SELN, *P*<0.001; COLEC11*TCCA-SEP vs. COLEC11*TCCA-SELP+SELN, *P*<0.01). Numbers in parentheses indicates absolute counts of sample size in each group. **SEP**: diagnosed with *S*. *haematobium* egg in urine; **SELP**: Negative for *S*. *haematobium* egg in urine but positive for anti-schistosoma total IgG; **SELN**: Negative for *S*. *haematobium* egg and anti-schistosoma total IgG.

The minor allele of exon8 variant rs7567833A (p.R216H) was significantly associated with increased CL-K1 serum levels (Fig. 3A and Fig. 3B). A gene dose-dependent effect on the distribution of serum CL-K1 levels was observed. Individuals with the COLEC11*TCCA haplotype had higher CL-K1 serum levels in both control groups (SELN: *P* = 0.01 and SELP+SELN: *P* = 0.0004) (Fig. 4), but such a trend was not observed in egg positive (SEP) individuals (S2 Fig). When only COLEC11*TCCA haplotypes were compared among the investigated groups, the SEP group had lower CL-K1 serum levels than the groups (SELP, SELP+SELN) (P<0.0001) (Fig. 5). The *COLEC11* haplotypes may further be classified as high expression (COLEC11*CCCG + COLEC11*TCCA) or as low expression (COLEC11*TCCG) haplotypes based on circulating serum CL-K1 levels observed in the control subgroups (Table 2). The low secretor haplotype (COLEC11*TCCG) was associated with *S*. *haematobium* infection (OR = 1.76, 95%CI = 1.15–2.70, *P* ^*corr*^ = 0.007) and the high secretor haplotypes were associated with decreased risk of infection (OR = 0.52, 95%CI = 0.33–0.79, *P* ^*corr*^ = 0.002).

## Discussion

Different immune strategies are employed by the host immune system to thwart an infection and the innate immune system plays a critical role in the clearance of some pathogens. Immune evasion from complement components is an important criterion for schistosomes to successfully establish an infection [14,39]. Lectin pathway proteins of the complement system are the first components to recognize the pathogen. These proteins can initiate a complement attack cascade independent of a specific antibody response [40]. Our previous studies have demonstrated that lectin proteins Ficolin-2 [37] and MBL [36] are involved in *S*. *haematobium* infections.

CL-K1 was first described in 2006 but relatively few studies only have looked at its role in infectious diseases. This study investigated the association between serum CL-K1 level and functional genetic variants in the *COLEC11* gene in urinary schistosomiasis. Our study suggests that a non-synonymous substitution p.R216H in the exon8 of *COLEC11* contributes to susceptibility to schistosomiasis. In particular, the major allele p.R216 increases the infection risk twofold compared to variant p.216H. Similar effects were also observed in different genetic models for the contribution of the respective p.R216H genotypes. It has been hypothesized that the p.R216H substitution increases the alpha helical propensity value that controls the protein stability and protein folding properties [41]. The p.R216H substitution is located in the carbohydrate recognition domain (CRD) of *COLEC11*, and therefore binding of the CRD with schistosome elements may be impaired. In addition, this particular variant rs7567833G/A (p.R216H) was reported to be under selective pressure [42] and was differentially distributed among a panel of 52 populations as described in HapMap and in the Human Genome Diversity Project–Centre d’Etude du Polymorphisme Humain (HGDP–CEPH) databases [43]. Also, the absence of LD with other genetic variants in proximity indicates selection. None of the promoter polymorphisms contributed to schistosomiasis susceptibility. In addition, the regulatory polymorphisms in the promoter region does not appear to play a role in CL-K1 expression as reported in another study [44].

Stratification of our study group based on reconstructed *COLEC11* haplotypes revealed that the frequency of the COLEC11*TCCG haplotype was significantly more frequent in the infection group than in controls, suggesting that individuals with these haplotypes had a higher risk of schistosomiasis. In addition, the COLEC11*TCCA haplotype harboring the rs7567833-A polymorphism occurred more frequently in healthy controls compared to the infection group, suggesting that individuals with this haplotype were protected from *S*. *haematobium* infection. Furthermore, when the SELP plus SELN controls were analyzed, significant differences were observed with the same haplotype, supporting the suggestion that the COLEC11*TCCA haplotype may help confer protection. The frequency of high CL-K1 expressing haplotypes was higher in controls than in SEP cases.

This study demonstrates that CL-K1 serum levels were higher in the control group compared with infected individuals, suggesting that high levels of CL-K1 might reduce the risk of *S*. *haematobium* infection. Similar to MBL and ficolins, CL-K1 could recognize and bind to specific glycoproteins on the surface of the pathogens [7,13]. In line with our earlier studies on Ficolin-2 [37] and MBL [36], we believe that CL-K1 serum levels may be down regulated during *S*. *haematobium* infection. Recent investigations in patients with disseminated intravascular coagulation (DIC) have shown that CL-K1 levels were significantly elevated [44]. The *COLEC11* rs7567833G/A (p.R216H) variant was observed to correlate with increased CL-K1 serum levels. In addition, the COLEC11*TCCA haplotype with the allele *p*.*216H* was associated with higher CL-K1 serum levels in healthy individuals. Inversely, COLEC11*TCCG with allele *p*.*R216* was associated with lower CL-K1 serum levels. These results substantiate that the variant in exon8 is a host genetic factor that may help protect against schistosomiasis. When the individuals with COLEC11*TCCA were analyzed for CL-K1 serum levels in the different patient groups, SEP individuals had lower levels than egg negatives, indicating that CL-K1 serum levels were modulated by infection.

CL-K1 has a collagen and a ligand binding domains, similar to MBL and ficolins. These structural domains bind specifically to pathogen-associated molecular patterns (PAMPs) on the surface of infectious agents [45]. We postulate that CL-K1, along with the MBL-associated serine proteases MASP-1/3 and MASP-2 initiate the complement lectin cascade to reduce *S*. *haematobium* infection. [20,30,34].

We have shown that allelic variants associated with increased CL-K1 levels may be a contributing protective host factor in schistosomiasis caused by *S*. *haematobium*. Furthermore, the variant in *p*.*R216H* in exon8 of the *COLEC11* gene is a host genetic factor associated with urinary schistosomiasis. Taken together, both *COLEC11* variants and CL-K1 serum levels are associated with the phenotype occurring after *S*. *haematobium* infection.

## Supporting Information

S1 Checklist STROBE checklist. (DOCX)

S1 Table Primer pairs and PCR program conditions utilized for screening the COLLEC11 gene. (DOCX)

S2 Table Distribution of screened COLEC11 variants in SELN Controls and its comparison with HapMap data of Yoruba ethnicity. (DOCX)

S1 Fig Linkage disequilibrium pattern of screened COLEC11 variants in SELN control group. Open white squares indicate a high degree of LD (D’ = 1) between pairs of markers. Numbers indicate the D’ value expressed as a percentile. The red square indicates pairs in strong LD with LOD scores ≥ 2; purple squares, D’ = 1 with LOD scores ≤ 1. A solid line outlines the haplotype block.
(TIF)

S2 Fig Distribution of CL-K1 serum levels (median values) with investigated COLEC11 haplotypes in SEP cases (SEP: diagnosed with S. haematobium egg in urine). P = 0.8 value illustrated in the figure is calculated by Kruskal-Wallis rank sum test. Numbers in parentheses indicates absolute counts of sample size in each group.
(TIF)
