# Supplementary material for: Lectin Complement Protein Collectin 11 (CL-K1) and Susceptibility to Urinary Schistosomiasis
Source: PLoS Negl Trop Dis. 2015 Mar 25;9(3):e0003647. doi: 10.1371/journal.pntd.0003647 (PMC4373859; doi:10.1371/journal.pntd.0003647)
Supplement: S1 Table — (DOCX) [file pntd.0003647.s002.docx]

**S1 Table: Primer pairs and PCR program conditions utilized for screening the *COLLEC11* gene.**

| Genomic Region | Primers | 5'-3' Sequence | Annealing Temperature(°C) | Fragment Size(bp) |
| --- | --- | --- | --- | --- |
| Promoter | COL11_Pro_F | TGGGCATGTTGTAGTTCGTGTTTTCA | 55 | 631 |
|  | COL11_Pro_R | TCTCCAACCCGAACATCCCAATTT |  |  |
| Exon1 | COL11_Ex1_F | CGGTGGACGCAGCGCAGACAG | 69.6 | 334 |
|  | COL11_Ex1_R | TTCCCATCCTCCTCGCTCAGCAG |  |  |
| Exon2 | COL11_Ex2_F | CCAGGCACCAGGAGGGCTACAC | 68.5 | 360 |
|  | COL11_Ex2_R | GGGCTTTGTGCTTTTCCGTGGA |  |  |
| Exon3 | COL11_Ex3_F | GCGGGGAGGGAGGTAGGGAGAG | 68.5 | 364 |
|  | COL11_Ex3_R | CCCAAGTCCCATGCCGTCTGC |  |  |
| Exon4 | COL11_Ex4_F | CCTGACCTCAAGAGATCCGCCTACCT | 68.5 | 377 |
|  | COL11_Ex4_R | TCCCAGCAAACCACGTCCATCTAGAA |  |  |
| Exon5 | COL11_Ex5_F | AGGAAGGAGGGCGGTCGGGTTA | 68.5 | 289 |
|  | COL11_Ex5_R | CACCGAATATAAATAAGAGCACCAGACGAG |  |  |
| Exon6 | COL11_Ex6_F | AATGGGGCTCCGGTACTTTGT | 58 | 220 |
|  | COL11_Ex6_R | AATCCTTGGACCCTTTATGCC |  |  |
| Exon7 | COL11_Ex7_F | GGGGTCCTCGCCTCTCTTCTGA | 68.5 | 229 |
|  | COL11_Ex7_R | GGGGGGCTCAGGACAGGGCT |  |  |
| Exon8 | COL11_Ex8_F | CTCACTTTTCAACCCTGCCTTAC | 59.8 | 780 |
|  | COL11_Ex8_R | AAGACCCCATTGCCACTATTT |  |  |
