# Supplementary material for: Lectin Complement Protein Collectin 11 (CL-K1) and Susceptibility to Urinary Schistosomiasis
Source: PLoS Negl Trop Dis. 2015 Mar 25;9(3):e0003647. doi: 10.1371/journal.pntd.0003647 (PMC4373859; doi:10.1371/journal.pntd.0003647)
Supplement: S2 Table — (DOCX) [file pntd.0003647.s003.docx]

**S2 Table: Distribution of screened *COLEC11 variants* in SELN Controls and its comparison with HapMap data of Yoruba ethnicity.**

| ***COLEC11*** | **(Position) rs ID- SNP** | **Amino Acid Change** | **Effect** | **Observed frequency** | **Hap Map frequency (Yoruba ethnicity)** |
| --- | --- | --- | --- | --- | --- |
| Promoter | (-676) rs1864480 C/T |  |  | 0.36 | 0.41 |
| Promoter | (-472) rs4849953 C/T |  |  | 0.25 | 0.21 |
| Promoter | (-469) rs6714770 C/G |  |  | 0.03 | no data |
| Promoter | (-276) rs3820897 C/T |  |  | 0.03 | 0.04 |
| Exon4 | (31012) rs10170348 A/G | K/K |  | 0.02 | 0.02 |
| Exon5 | (42539) rs17017791 C/T | I/I |  | 0.12 | 0.13 |
| Exon7 | (48429) rs34351135 A/G | K/K |  | 0.11 | no data |
| Exon7 | (48435) rs34436491 C/T | I/I |  | 0.03 | no data |
| Exon 8 | (48912) rs7567833 G/A | R/H | α-Helix Propensity | 0.32 | 0.19 |
