# Supplementary figures and images for: Lectin Complement Protein Collectin 11 (CL-K1) and Susceptibility to Urinary Schistosomiasis
Source: PLoS Negl Trop Dis. 2015 Mar 25;9(3):e0003647. doi: 10.1371/journal.pntd.0003647 (PMC4373859; doi:10.1371/journal.pntd.0003647)

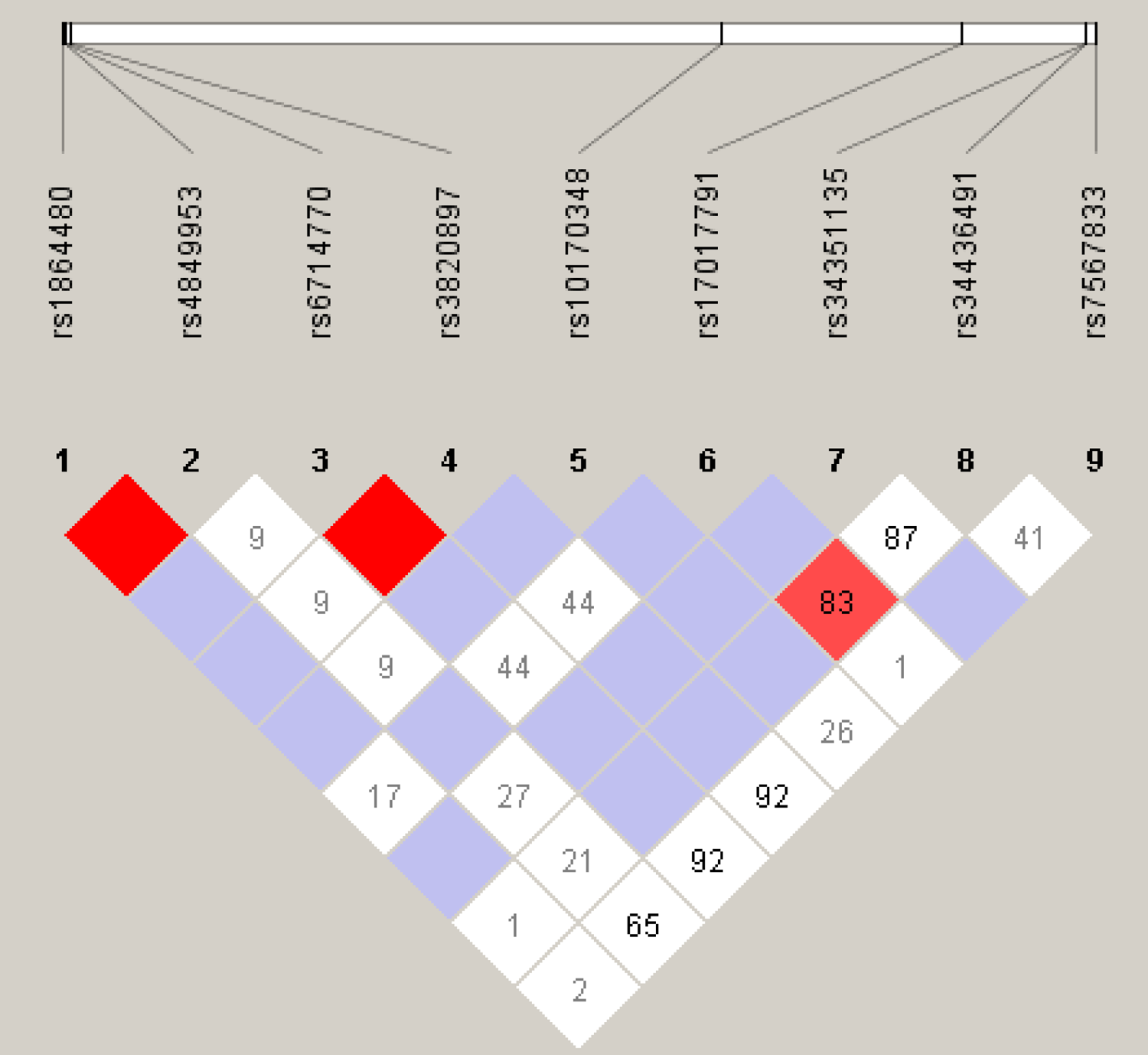

Supplement: S1 Fig — Open white squares indicate a high degree of LD (D’ = 1) between pairs of markers. Numbers indicate the D’ value expressed as a percentile. The red square indicates pairs in strong LD with LOD scores ≥ 2; purple squares, D’ = 1 with LOD scores ≤ 1. A solid line outlines the haplotype block. (TIF) [file pntd.0003647.s004.tif]

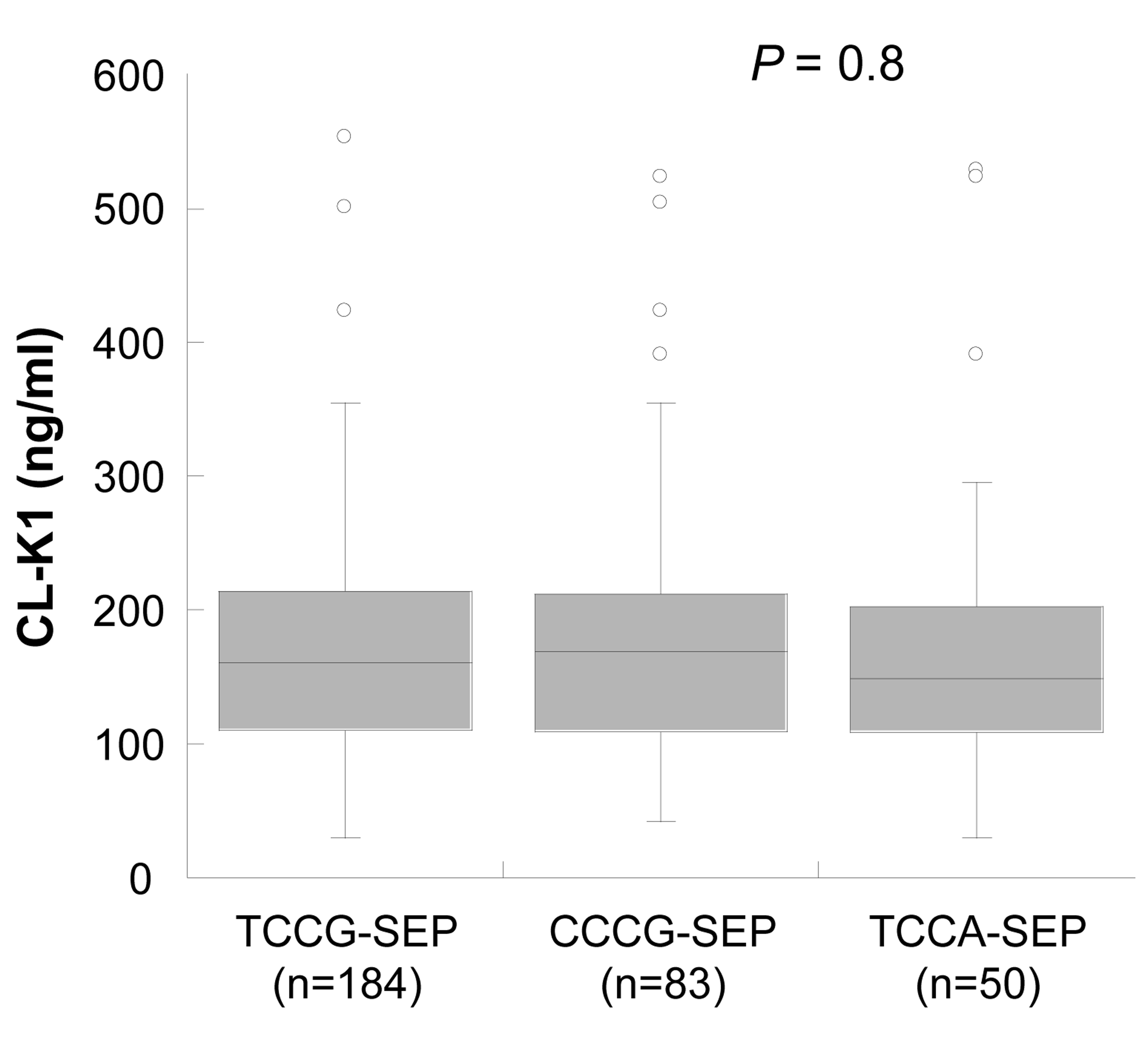

Supplement: S2 Fig — P = 0.8 value illustrated in the figure is calculated by Kruskal-Wallis rank sum test. Numbers in parentheses indicates absolute counts of sample size in each group. (TIF) [file pntd.0003647.s005.tif]
